# Expanding the use of salivary cortisol as a non-invasive outpatient test in the dynamic evaluation of suspected adrenal insufficiency

**DOI:** 10.1530/EC-23-0004

**Published:** 2023-03-28

**Authors:** Sarah Ying Tse Tan, Hong Chang Tan, Ling Zhu, Lih Ming Loh, Dawn Shao Ting Lim, Du Soon Swee, Yoke Ling Chan, Huee Boon Lim, Shiau Lee Ling, En Jun Ou, Wynn Ee Teo, Xiao Ping Zhang, Hui Fen Goh, Peng Chin Kek

**Affiliations:** 1 Department of Endocrinology, Singapore General Hospital, Singapore; 2 Department of Speciality Nursing, Singapore General Hospital, Singapore

**Keywords:** adrenal insufficiency, synacthen test, ACTH stimulation test, salivary cortisol

## Abstract

**Background:**

Adrenal insufficiency (AI) is potentially life-threatening, and accurate diagnosis is crucial. The first-line diagnostic test, the adrenocorticotrophic hormone (ACTH) stimulation test, measures serum total cortisol. However, this is affected in states of altered albumin or cortisol-binding globulin levels, limiting reliability. Salivary cortisol reflects free bioactive cortisol levels and is a promising alternative. However, few studies are available, and heterogenous methodologies limit applicability.

**Methods:**

This study prospectively recruited 42 outpatients undergoing evaluation for AI, excluding participants with altered cortisol-binding states. Serum (immunoassay) and salivary (liquid chromatography tandem mass spectrometry) cortisol levels were sampled at baseline, 30 min, and 60 min following 250 µg synacthen administration. AI was defined as a peak serum cortisol level <500 nmol/L in accordance with guidelines.

**Results:**

The study recruited 21 (50%) participants with AI and 21 without AI. There were no significant differences in baseline characteristics, blood pressure, or sodium levels between groups. Following synacthen stimulation, serum and salivary cortisol levels showed good correlation at all timepoints (*R*^2^ = 0.74,* P* < 0.001), at peak levels (*R*^2^ = 0.72, *P* < 0.001), and at 60 min (*R*^2^ = 0.72, *P* < 0.001). A salivary cortisol cut-off of 16.0 nmol/L had a sensitivity of 90.5% and a specificity of 76.2% for the diagnosis of AI.

**Conclusion:**

This study demonstrates a good correlation between serum and salivary cortisol levels during the 250 µg synacthen test. A peak salivary cortisol cut-off of 16.0 nmol/L can be used for the diagnosis of AI. It is a less invasive alternative to evaluate patients with suspected AI. Its potential utility in the diagnosis of AI in patients with altered cortisol-binding states should be further studied.

## Background

Adrenal insufficiency (AI) is associated with increased mortality, making accurate and timely diagnosis a key to improving patient outcomes (1, 2).

Several tests have been developed to diagnose AI, but in the clinical setting, the adrenocorticotrophic hormone (ACTH) stimulation test (AST) is the most commonly used (3). During the AST, 250 µg of synthetic ACTH was administered intravenously or intramuscularly, and serum cortisol is drawn at baseline, 30 min, and 60 min after administration (3). AI is diagnosed when the peak cortisol level is below 500 nmol/L at 30 or 60 min (3). However, the AST is a labour-intensive procedure, requiring the expertise of skilled personnel (nurses or phlebotomist). Consequently, the AST is often performed in the outpatient endocrine unit by specialist nurses, which may limit the availability of an important diagnostic test, especially in centres with none or few trained personnel.

Another limitation of the AST is that most laboratories measure serum total cortisol concentrations and do not differentiate between the bound and free forms. Cortisol in blood exists in two forms: cortisol that is bound to carrier molecules (cortisol-binding globulin and albumin) and cortisol that is unbound, i.e. free cortisol. Free cortisol is the biologically active form, and it is the serum concentration of free cortisol that is clinically and physiologically relevant (4). In healthy individuals, the ratio of bound (90%) and unbound (10%) cortisol is fairly consistent, and the total cortisol concentration is adequate to diagnose AI (5). However, there are certain medical states and medications that alter cortisol binding or the ratio of bound to unbound cortisol, thus complicating the diagnosis of AI. This occurs in patients with critical illness, chronic liver disease, and hypoalbuminaemia, which reduce cortisol binding, as well as pregnancy and oral oestrogen therapy, which increase cortisol binding (6, 7, 8, 9). In these cases, the measurement of total cortisol would not accurately reflect the free, bioactive component and may lead to misdiagnosis of AI.

Indeed, studies in subjects with critical illness and chronic liver disease have shown that there is a significant discrepancy between total and free cortisol levels, with total cortisol levels underestimating the cortisol response (10, 11). However, assays for direct measurement of serum-free cortisol concentrations are not widely available in the clinical setting, and calculations of the free cortisol index are limited by the measurement of cortisol-binding globulin and do not account for changes in binding affinity or albumin concentrations (12, 13).

Salivary cortisol is an alternative method to measure free cortisol. It has been shown that the concentrations of free cortisol in the saliva are at an equilibrium with free cortisol concentrations in the serum, and this relationship is independent of the rate of saliva production (14). It is already in use as one of the diagnostic tests for Cushing’s syndrome (15). Its role in the diagnosis of AI is promising, with several studies exploring the measurement of salivary cortisol following AST (16, 17, 18, 19, 20, 21, 22, 23, 24, 25, 26, 27, 28, 29, 30, 31, 32, 33). These studies have demonstrated a good correlation between serum and salivary cortisol levels following AST (22, 23). However, the salivary cortisol cut-off levels for the diagnosis of AI derived from these studies varied widely from 8.3 to 39.5 nmol/L, likely due to the great heterogeneity in the study populations, methods, timing of samples, dose of synthetic ACTH, and assays used (Supplementary Table 1, see the section on supplementary materials given at the end of this article) (34). In addition, the collection of salivary cortisol is non-invasive and overcomes the need for skilled personnel for repeated blood draws.

Our study had two aims: (1) to assess the correlation between serum and salivary cortisol levels following AST in subjects with normal cortisol-binding states and (2) to determine the optimum salivary cortisol cut-off level for the diagnosis of AI and its associated test performance.

## Methods

### Subjects

We recruited subjects from the endocrine testing clinic at the Department of Endocrinology at our tertiary centre (August 2020 to January 2022) who were planned for AST for the evaluation of suspected AI. Exclusion criteria consisted of conditions that may affect cortisol binding, including liver cirrhosis, advanced chronic kidney disease (estimated glomerular filteration rate < 30 mL/min/1.73m^2^ or end-stage kidney failure on renal replacement therapy), pregnancy, oral contraceptive medication use, active malignancies or eating disorders, or weight loss of >10% over the past 3 months. Subjects with recent dental procedures or oral bleeding and those unable to follow instructions or provide informed consent were also excluded. This study was approved by the relevant Institutional Review Board (SingHealth Centralised Institutional Review Board 2020/2434), and all subjects provided written informed consent.

### ACTH stimulation test

Eligible patients underwent the AST, which was conducted in the outpatient endocrine unit between 08:00 and 10:00 by specialised nursing staff. Subjects on chronic hydrocortisone replacement were instructed to omit the medication on the morning of the test. They were also instructed not to eat, drink, or brush their teeth for 15 min prior to specimen collection and throughout the duration of the test.

An intravenous cannula was inserted followed by the simultaneous collection of baseline plasma and saliva samples (0-min sample). Blood was collected in an EDTA tube and analysed upon receipt by the laboratory. Saliva specimens were collected using the SARSTEDT Salivette®. Subjects were instructed to place the SARSTEDT Salivette® into their mouth for 2 min to obtain at least 1.5 mL of saliva per specimen. About 250 µg of ACTH (Synacthen®, Novartis) was then injected intravenously, followed by the simultaneous collection of serum and salivary cortisol samples at 30 and 60 min.

AI was diagnosed if peak serum cortisol levels failed to reach 500 nmol/L (3). Post-AST serum cortisol results were reviewed, and patients were then assigned to the group in the sequence that they presented. Salivary cortisol specimens were sent off for the first 21 subjects with AI and the first 21 without AI.

### Laboratory assays

Serum cortisol was measured using the Beckman Coulter UniCel DxI 800 Access Immunoassay Systems. The detection limit was 11 nmol/L. The assay exhibited a total imprecision of <12% at approximately 138 nmol/L and <10% for higher concentrations of cortisol (35).

Salivary cortisol measurement was performed at the Mayo Medical Laboratories. Salivary cortisol was extracted from the specimen using online turbulent flow high-performance liquid chromatography and analysed by liquid chromatography tandem mass spectrometry (LC-MS/MS) using multiple reaction monitoring in positive mode (36). The detection limit was 0.11 nmol/L. The intra-assay coefficient of variation was 7.2% at 3.0 nmol/L, and the inter-assay coefficient of variation was 5.8% at 1.4 nmol/L (36).

### Statistical analysis

We estimated that 21 patients with AI and 21 without AI will be adequate to achieve a receiver operating characteristic curve (ROC) area under the curve (AUC) of 0.9 (confidence interval (CI) 0.8–1). Statistical analysis was performed using SPSS Statistics 21.0 (IBM) and GraphPad Prism Version 9.3.0 for Windows (GraphPad Software). Baseline characteristics and descriptive statistics were computed and expressed as mean ± β standard deviation (continuous variables) and frequency (categorical variables). A *P* value of < 0.05 indicated statistical significance.

Peak serum and salivary cortisol levels were defined as the highest value across the three timepoints. Linear regression analysis was used to examine the relationship between free cortisol concentrations in serum and saliva in all samples at 60 min and the peak value.

ROC curves were generated to identify the optimal salivary cortisol cut-off value for the diagnosis of AI. The diagnostic performance of salivary cortisol during 0, 30, 60 min, and peak value was investigated using the ROC analysis. Youden’s index (sensitivity (%) + specificity (%) – 100) was used to estimate the optimal peak salivary cortisol cut-off value to diagnose AI.

## Results

**Figure 1:**
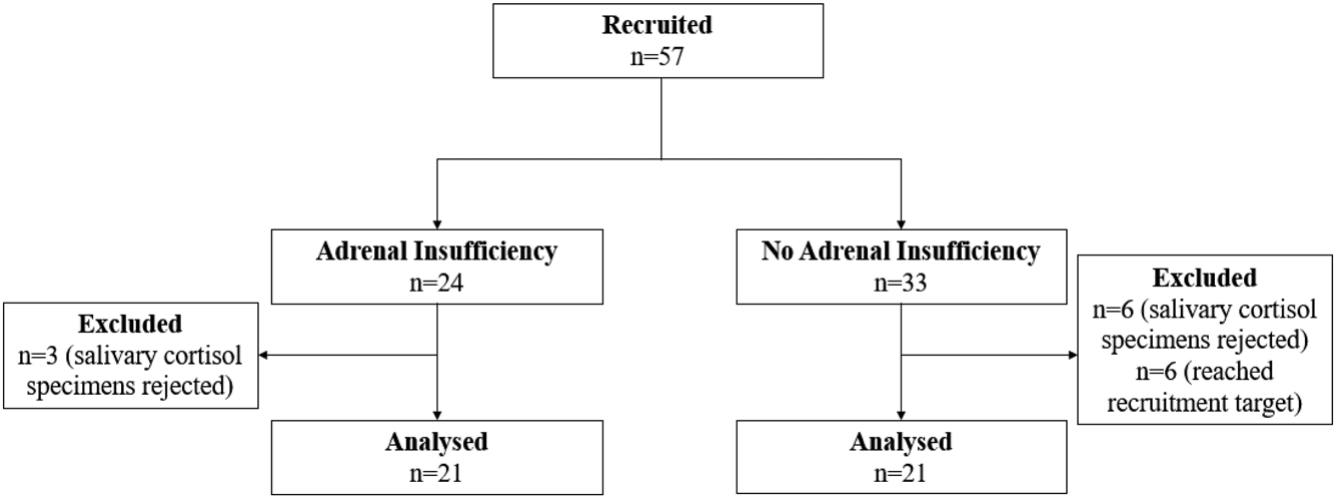
Consort diagram.

A total of 57 subjects were recruited into the study: 24 with AI and 33 without AI (Fig. 1). Three subjects from the AI arm and six subjects from the arm without AI were excluded from the analysis as their salivary cortisol specimens were rejected by the laboratory due to technical reasons. Salivary cortisol was not analysed for six subjects from the arm without AI as the recruitment target (*n* = 21) had been reached for that group.

The baseline characteristics and laboratory results of the subjects with and without AI are reflected in Table 1. The aetiologies of AI were due to exogenous glucocorticoid use in 16, primary AI in 2, hypopituitarism in 1, and post-adrenalectomy for adrenal Cushing’s syndrome in 1. Thirteen were prescribed regular hydrocortisone replacement, 7 were given standby hydrocortisone to be taken on sick days, and 1 was not prescribed hydrocortisone. Aside from an older average age amongst the subjects with AI (AI = 62.2 ± 14.6 years old; no AI = 51.1 ± 16.4 years old), there was no significant difference between the two groups. The average body mass index, blood pressure, sodium, and albumin normal were normal.

**Table 1:** Baseline characteristics and laboratory results.

Variable	Adrenal insufficiency *n* = 21	No adrenal insufficiency *n* = 21	*P*-value
Male (%)	16 (68.2%)	10 (45.5%)	
Age (years)	62.2 ± 14.6	51.1 ± 16.4	0.026
BMI (kg/m^2^)	26.2 ± 5.5	26.7 ± 9.6	0.831
SBP (mmHg)	130.4 ± 16.1	123.8 ± 18.0	0.220
DBP (mmHg)	71.4 ± 9.4	67.3 ± 10.9	0.200
Creatinine	78.4 ± 22.4	74.1 ± 22.5	0.540
Sodium (mmol/L)	139.5 ± 2.3	139.3 ± 2.8	0.859
Potassium (mmol/L)	4.2 ± 0.5	4.2 ± 0.3	0.459
Albumin (g/L)	39.8 ± 4.1	40.3 ± 3.2	0.677

Values are given as mean ± standard deviation or frequency (%).

BMI, body mass index; DBP, diastolic blood pressure; SBP, systolic blood pressure.

Amongst the 42 subjects, 41 (98%) reached the peak salivary cortisol level at 60 min, while 1 subject reached the peak salivary cortisol level at 30 min. Subjects without AI had significantly higher 30-min, 60-min, and peak serum and salivary cortisol levels compared with those without AI (Table 2, *P*-values all < 0.001). In the subjects with AI, the mean serum cortisol levels were 232.6 ± 92.6 nmol/L at baseline, 369.2 ± 107.6 nmol/L at 30 min, 393.6 ± 105.4 nmol/L at 60 min, and the mean peak level was 397.6 ± 107.0 nmol/L. The corresponding salivary cortisol levels were 3.4 ± 2.1 nmol/L (0 min), 8.0 ± 5.7 nmol/L (30 min), 11.0 ± 7.7 (60 min), and 11.0 ± 7.7 (peak). There was no significant difference in baseline serum or salivary cortisol levels between the two groups (serum cortisol: no AI = 309.6 ± 120.6 nmol/L; AI = 232.6 ± 92.6 nmol/L; salivary cortisol: no AI = 4.6 ± 3.0 nmol/L; AI = 3.4 ± 2.1 nmol/L; *P* = NS).

**Table 2:** Serum and salivary cortisol concentrations during AST.

Timepoint	0 min		30 min		60 min		Peak	
Cortisol	Serum (nmol/L)	Salivary (nmol/L)	Serum (nmol/L)	Salivary (nmol/L)	Serum (nmol/L)	Salivary (nmol/L)	Serum (nmol/L)	Salivary (nmol/L)
Total (*n* = 42)	271.1 ± 113.1	4.0 ± 2.6	473.3 ± 162.0	13.3 ± 9.2	527.0 ± 185.3	20.1 ± 14.8	529.2 ± 184.1	20.1 ± 14.8
Adrenal insufficiency (*n* = 21)	232.6 ± 92.6	3.4 ± 2.1	369.2 ± 107.6	8.0 ± 5.7	393.6 ± 105.4	11.0 ± 7.7	397.6 ± 107.0	11.0 ± 7.7
No adrenal insufficiency (*n* = 21)	309.6 ± 120.6	4.6 ± 3.0	577.4 ± 139.5	18.6 ± 8.9	660.5 ± 147.9	29.2 ± 14.7	660.5 ± 147.9	29.2 ± 14.7

*P-*values < 0.01 for adrenal insufficiency vs no adrenal insufficiency groups for serum and salivary cortisol levels at 30 min, 60 min, and peak levels. Values reflected are mean ± standard deviation.

### Relationship between serum and salivary cortisol after AST

**Figure 2:**
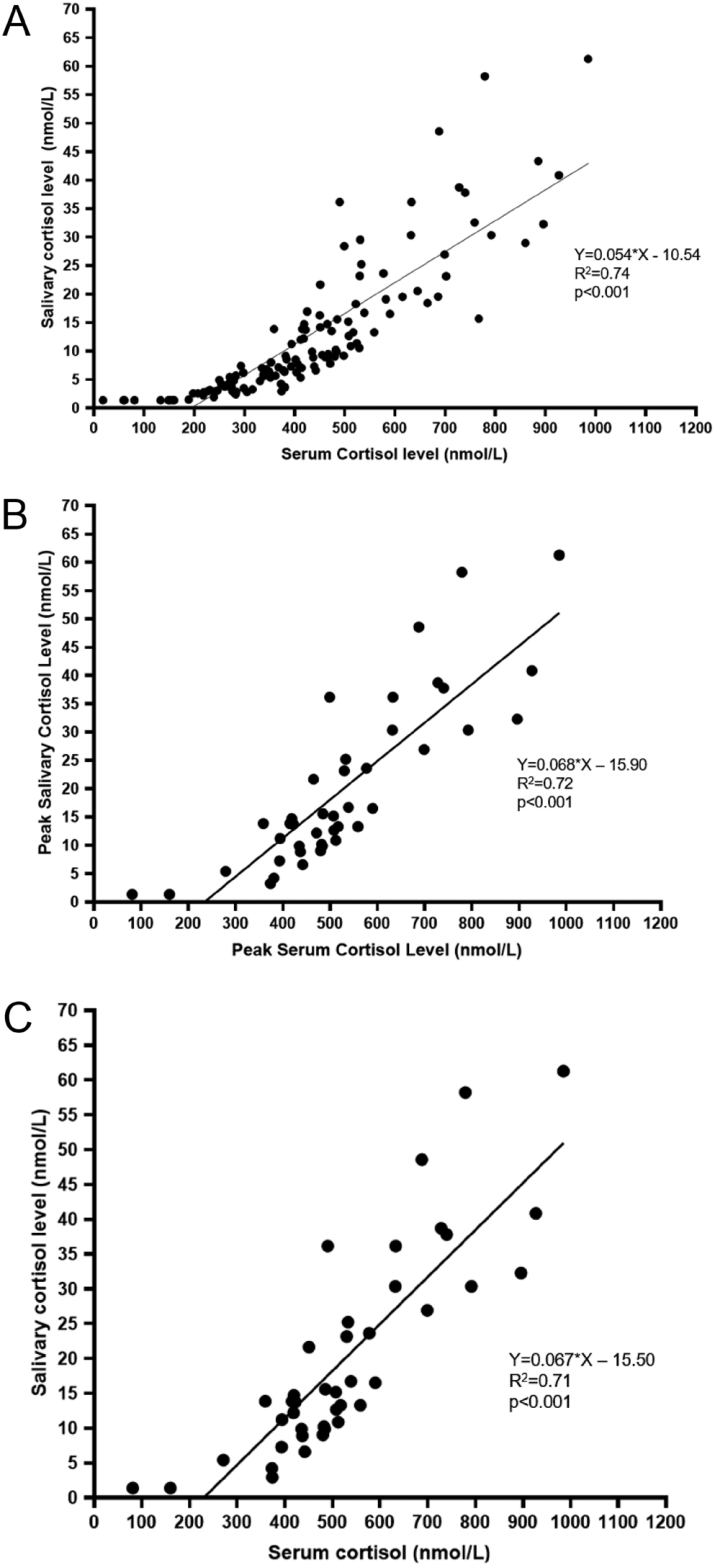
Relationship between serum and salivary cortisol levels after AST. (A) All serum and salivary cortisol levels. (B) Peak serum and salivary cortisol levels. (C) Sixty-minute serum and salivary cortisol levels.

Serum and salivary cortisol levels showed good levels of correlation across all timepoints (Fig. 2A, *R*^2^ = 0.74, *P* < 0.001), at peak levels (Fig. 2B, *R*^2^ = 0.72, *P* < 0.001), and at 60 min (Fig. 2C, *R*^2^ = 0.72, *P* < 0.001). Although the group with AI had a higher mean age compared with those without AI, the correlation between peak serum and salivary cortisol remained statistically significant even after controlling for age (*R*^2^= 0.83, *P* < 0.001). The relationship between serum and salivary cortisol was also examined using a non-linear model, which yielded similar results.

### ROC analysis

**Figure 3:**
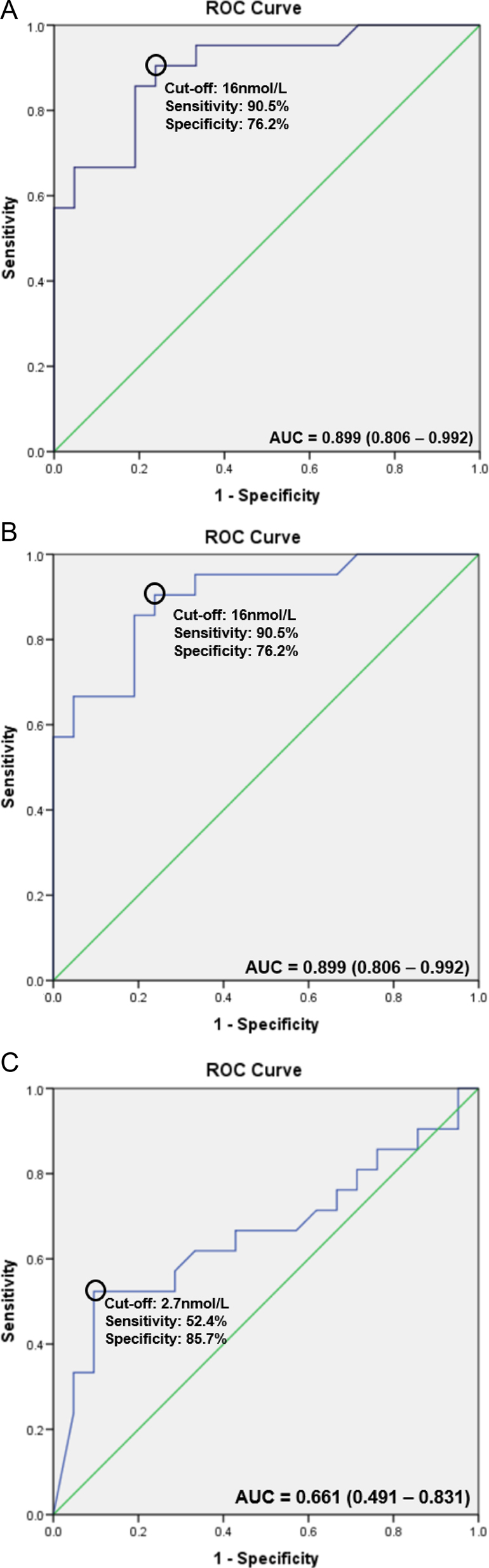
ROC analysis for diagnosis of adrenal insufficiency using salivary cortisol. (A) Peak salivary cortisol. (B) Sixty-minute salivary cortisol. (C) Zero-minute salivary cortisol.

The ROC curve for peak salivary cortisol levels had an AUC of 0.899 (95% CI: 0.806–0.992, Fig. 3A). The best peak salivary cortisol cut-off level derived using Youden's index was 16.0 nmol/L, with a sensitivity of 90.5% and a specificity of 76.2%. AUC for 60-min salivary cortisol levels was 0.899 (95% CI: 0.806–0.992, Fig. 3B). The positive predictive value was 71.5% and the negative predictive value was 88.9%.

**Figure 4:**
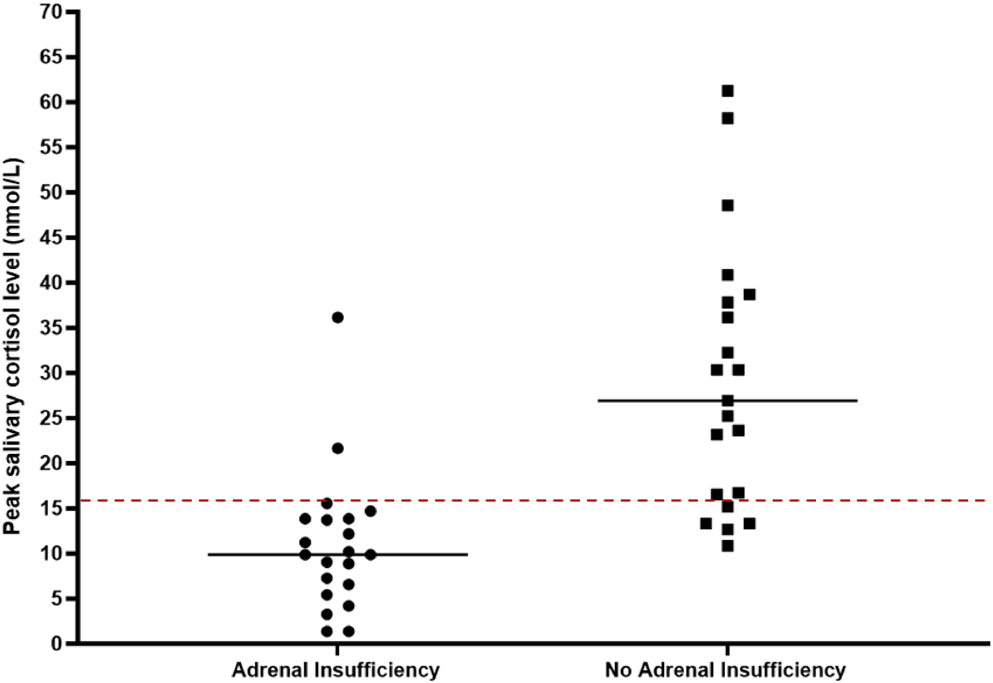
Scatterplot.

Figure 4 depicts the scatterplot of subjects with and without AI when a peak salivary cortisol cut-off of 16.0 nmol/L is used.

Compared with peak salivary cortisol levels, baseline salivary cortisol levels had lower diagnostic accuracy for the diagnosis of AI, with an AUC of 0.661 (95% CI: 0.491–0.831, Fig. 3C). The best 0-min salivary cortisol cut-off derived using Youden’s index was 2.7 nmol/L, with a sensitivity of 52.4% and a specificity of 85.7%.

## Discussion

A misdiagnosis of AI can lead to under- or overtreatment with glucocorticoid replacement therapy. The standard diagnostic method using the AST works well in most patients but is limited by the use of serum cortisol for diagnosis as well as the need for trained personnel for multiple blood draws. As salivary cortisol reflects the free cortisol component in the serum, it can potentially overcome the confounding effects from changes in cortisol-binding dynamics due to physiological or pathological reasons. Moreover, the sample collection for salivary instead of serum cortisol is less invasive.

In this study, we found that salivary cortisol explains more than 70% variance in the concentration of total serum cortisol, and the significant correlation between salivary cortisol holds true for the samples collected at baseline, 30 min, and 60 min following AST. The correlation coefficient in our study (*R* ^2^ = 0.72) was similar to what has been described in the literature (*R* = 0.75 reported by Kim *et al.*; *R* = 0.84 reported by Elder *et al.*) (18, 23). Although one study reported better results when an exponential model was used to describe the relationship between serum and salivary cortisol (*R* ^2^ = 0.83 vs *R* ^2^ = 0.65) (22), we obtained comparable results by using a linear model.

A peak salivary cortisol cut-off level of 16.0 nmol/L had good sensitivity and specificity for the diagnosis of AI amongst subjects with normal cortisol-binding states. Peak salivary cortisol cut-off levels of 13–16 nmol/L following AST have been described in several studies. Cornes *et al.* included 36 subjects with suspected AI who underwent an intravenous 250 µg AST (16). Using a peak serum cortisol of <550 nmol/L to diagnose AI, a peak salivary cortisol cut-off of ≥15 nmol/L was derived. Langelaan *et al.* investigated 129 subjects with suspected AI similarly undergoing a 250 µg intravenous AST (19). AI was defined as a peak serum cortisol of <550 nmol/L on the Roche I assay or <420 nmol/L on the Roche II assay. A 60-min salivary cortisol cut-off level of 15.6 nmol/L was derived for the diagnosis of AI. Kim *et al.* studied 120 subjects with suspected AI who underwent a 250 µg intravenous AST (23). AI was defined as a peak serum cortisol <496.8 nmol/L. A peak salivary cortisol cut-off of 13.2 nmol/L had a sensitivity of 90.7% and a specificity of 94.1% for the diagnosis of AI. These studies included similar participants (suspected AI) and methodologies (250 µg intravenous AST with cortisol levels measured at 0, 30, and 60 min) as our study. However, the differences in serum and salivary cortisol assays, as well as the varying definitions of AI using serum cortisol, could account for the small differences in the salivary cortisol cut-off level derived (37).

In addition to studies that included ASTs, a study by Karpman *et al.* investigated the role of salivary cortisol for the diagnosis of AI during the insulin tolerance test, the gold standard test for the diagnosis of AI. AI was defined by a peak serum cortisol level <500 nmol/L. It found that a salivary cortisol cut-off level of 13.3 nmol/L had a sensitivity of 87.5% and specificity of 89.3% for the diagnosis of AI (38). The cut-off level derived was only slightly lower than that derived in the present and previous studies discussed above.

While peak salivary cortisol levels had a good sensitivity and specificity for the diagnosis of AI, the mean baseline early morning (0-min) salivary cortisol levels were not significantly different between patients with and without AI and had lower diagnostic accuracy (sensitivity 52.4%). This is unsurprising, given that early morning serum cortisol levels have not been shown to have good sensitivity for the diagnosis of AI (39). Restituto *et al.* showed that morning salivary cortisol levels had low sensitivity (33%) and specificity (20%) for the diagnosis of AI, and the overall performance was similar to that of morning serum cortisol levels (40). Thus, AST would still be required for the diagnosis of AI regardless of whether serum or salivary cortisol levels are measured.

In our study, the peak salivary cortisol level occurred at 60 min in 98% of subjects, and the salivary cut-off levels derived for both peak and 60-min cortisol levels were the same (16.0 nmol/L). A peak salivary cortisol level at 60 min has also been described in other studies (18, 23). Thus, it may be possible to measure the 60-min salivary cortisol level alone (without the baseline and 30-min samples), thus simplifying the test and reducing the cost further. A further less invasive option would be to pair this with the use of intramuscular synthetic ACTH, which has also been described in studies with salivary cortisol (29). This would be a useful option in patients with difficult venous access, and more studies in this area would be helpful.

A strength of this study is the fact that subjects with abnormal cortisol-binding states were excluded, as serum cortisol levels may not be accurate for the diagnosis of AI amongst these subjects. To achieve this study’s objectives, the study population was kept homogenous and intentionally excluded patients with conditions that may affect cortisol binding, to minimise the presence of confounding factors that may affect serum cortisol levels. However, as these subjects were not assessed in this study, further studies would be required to investigate the role of salivary cortisol for the diagnosis of AI in subjects with altered cortisol-binding states and to validate whether the same diagnostic threshold can be used.

Another strength was that this study aimed to be reflective of real-life clinical practice in the diagnosis of AI. First, AI was defined based on AST rather than the insulin tolerance test. The latter is associated with multiple risks and is largely limited to research settings, and AST has been validated against it for the diagnosis of primary AI (3). Secondly, like most other studies, serum cortisol was measured using an immunoassay. Ideally, LC-MS/MS is the gold standard for the measurement of serum cortisol. Significant variability between different modern cortisol immunoassays has been described, including the suggestion of different diagnostic thresholds for AI based on different immunoassays used (37, 41). However, LC-MS/MS is not widely available in clinical settings, and the use of immunoassay is more reflective of clinical practice. AI was diagnosed if the peak serum cortisol levels failed to reach 500 nmol/L at 30 or 60 min in accordance with societal clinical practice guidelines (3).

Thirdly, salivary cortisol measurement was performed using LC-MS/MS, and salivary cortisol was collected using a salivette, which has been shown to be a reliable method even at low amounts of saliva or cortisol levels. Poll *et al.* demonstrated that salivary cortisol collection using salivettes was a better predictor of total and free serum cortisol compared to the passive drooling method and that it was the preferred method by participants and staff (42).

However, there are also several limitations. First, despite standardising the procedure for salivary cortisol collection, several (9) subjects had their salivary cortisol specimens rejected by the laboratory due to ‘insufficient specimens’. Amongst these patients, one had a medical history of Sjogren's syndrome, one had a previous stroke with significant neurological deficit, and two had hypothyroidism. These conditions may affect the production or collection of saliva samples (43, 44). This had not been reported in other studies looking at the role of salivary cortisol in AST. It is possible that the frequency of saliva samples required (three within an hour) could affect the adequacy of saliva collected in the subsequent samples. If the salivary cortisol collection can potentially be reduced to one (at the 60-min mark), this may mitigate the issue. However, further assessment is required before introducing this as a clinical test, and subjects with altered saliva production may need to be excluded. Secondly, the number of participants included in this study was smaller compared to other existing studies.

Lastly, salivary cortisone was not measured in this study. Salivary cortisol is converted to salivary cortisone by the 11β-hydroxysteroid dehydrogenase 2 enzyme that is present in high levels in the saliva (45). Several recent studies have explored its role in the diagnosis of AI. Elder *et al.* described a strong correlation between serum cortisol and salivary cortisol following AST (18). Debono *et al.* also described that a waking salivary cortisone cut-off value of 17 nmol/L had good sensitivity and specificity for the diagnosis of AI (46). Further studies to investigate and validate these diagnostic thresholds for the diagnosis of AI would be helpful.

In conclusion, salivary cortisol is a promising alternative to serum cortisol in the diagnosis of AI following AST. Serum and salivary cortisol levels demonstrated a good correlation during the AST. A peak salivary cortisol cut-off of 16.0 nmol/L measured using LC-MS/MS has a good sensitivity and specificity for the diagnosis of AI. Not only is salivary cortisol potentially more accurate than serum cortisol in the diagnosis of AI, it is also a simple and less invasive alternative. The use of salivary cortisol can potentially improve the accuracy and accessibility of the AST for the diagnosis of AI. Further studies would be helpful to assess and validate its role in subjects with altered cortisol-binding states.

## Supplementary Material

Supplementary Table 1: Literature Review of Studies Assessing Salivary Cortisol in AST

## Declaration of interest

There are no conflicts of interest to disclose.

## Funding

This work was funded by Pitch For Funds Award, SingHealth Duke-National University of Singapore Medicine Academic Clinical Programme Research Support Programme Grant (grant number 03/FY2018/P1/13-A28_FY2020PFF03).

## Data availability statement

The data that support the findings of this study are available from the corresponding author upon reasonable request.
