# Supplementary material for: Expanding the use of salivary cortisol as a non-invasive outpatient test in the dynamic evaluation of suspected adrenal insufficiency
Source: Endocr Connect. 2023 Mar 28;12(4):e230004. doi: 10.1530/EC-23-0004 (PMC10083658; doi:10.1530/EC-23-0004)
Supplement: Supplementary Table 1: Literature Review of Studies Assessing Salivary Cortisol in AST [file supplementary_table_1.pdf]

| Supplementary Table 1: Literature Review of Studies Assessing Salivary Cortisol in AST | | | | | | |
|---|---|---|---|---|---|---|
| **Study** | **Participants** | **ACTH Stimulation Test (AST) Protocol** | **Serum Cortisol Assay and Cut-off** | **Salivary Cortisol Assay** | **Relationship between Serum and Salivary Cortisol** | **Salivary Cortisol Cut-off** |
| **Albert, 2019.**[30] | 45 healthy volunteers, 39 AI, 39 decompensated cirrhosis | • IV 250mcg AST<br>• Serum and salivary cortisol measured at 0, 30, 60, 90 minutes | Roche immunoassay; Serum cortisol > 248 nmol/L at 0 minutes, > 497 nmol/L at 60 minutes, or increase between 0 minute and 60 minute samples > 248 nmol/L | Electrochemi-lumiscence assay | R=0.79, *P*<0.01 | 0 minute < 2.2 nmol/L<br>60 minutes < 39.5 nmol/L<br>Rise in salivary cortisol < 27.6 nmol/L<br>(Sn/Sp not reported) |
| **Chao, 2019.**[31] | 21 healthy children | • IV or IM 250mcg AST<br>• Serum and salivary cortisol measured at 0 and 45 minutes | Immunoassay; Complete AI: peak serum cortisol < 275 nmol/L<br>Partial AI: peak serum cortisol 275 - 497 nmol/L | LC-MS | $R^2$=0.96 | Peak salivary cortisol >13.8 nmol/L<br>(sensitivity 100%, specificity 100%) |
| **Contreras, 2004.**[26] | 21 healthy volunteers, 19 AI | • IM 12.5mcg, 25mcg, 250mcg AST<br>• Serum cortisol measured at 0 and 30 minutes<br>• Salivary cortisol measured at 0, 15, 30, 45, 60, 75, 90, 105, 120 minutes | Radio-immunoassay; Serum cortisol ≥ 552nmol/L | Radio-immunoassay | R=0.099, *P*=0.665 | Salivary cortisol >20 nmol/L 30 minutes after AST (based on healthy subjects; Sn/Sp not reported) |
| **Cornes, 2015**[16] | 36 suspected AI | • AST performed in afternoon | Roche immunoassay | LC-MS | $R^2$=0.6037 | Peak salivary cortisol ≥15nmol/L |

| | | | | | | |
|---|---|---|---|---|---|---|
| | | • IV 250mcg AST<br>• Serum and salivary cortisol measured at 30 and 60 minutes | Peak serum cortisol ≥ 550nmol/L | | | (Sn/Sp not reported) |
| **Deutschbein, 2009.**[25] | 21 healthy volunteers, 55 AI | • IV 250mcg AST<br>• Serum and salivary cortisol measured at 0, 30, 60, 90, 120 minutes | Centaur immunoassay; ITT peak cortisol ≥ 500 nmol/L | Radio-immunoassay | R=0.88 *P*<0.001 | Salivary cortisol at 30 minutes: >32.5 nmol/L (sensitivity 72%; specificity 86%) |
| **Dušková, 2016.**[17] | 15 healthy men | • IV 1, 10, 250mcg AST<br>• ITT | LC-MS | LC-MS | NA | Peak salivary cortisol ≥12.5 nmol/L (healthy subjects) |
| **Elbuken, 2014.**[32] | 44 healthy volunteers | • IV 1, 250mcg AST<br>• Serum and salivary cortisol measured at 0, 30, 60 minutes (for 1mcg AST) and 0, 30, 60, 90 minutes (for 250mcg AST) | Radio-immunoassay | Immunoassay | NA | Peak salivary cortisol ≥ 5.8 nmol/L (1mcg AST) or ≥ 23.4 nmol/L (250mcg AST) (healthy subjects) |
| **Elder, 2018.**[18] | 35 healthy volunteers | • IV 1, 250mcg AST<br>• 1mg dexamethasone given the night before and morning of AST<br>• Serum and salivary cortisol measured from 0 to 120 minutes | Abbott Architect immunoasasy | LC-MS | R=0.82, *P*<0.001 | NA |
| **George, 2020.**[29] | 67 suspected AI | • IM 30u porcine corticotrophin<br>• Salivary cortisol measured at 0, 60, 120 minutes | Roche II immunoassay; peak serum cortisol ≥ 500 nmol/L | Electrochemi-luminescence | R=0.88, *P*<0.001 | Peak salivary cortisol:<br>60 mins: >18.5 nmol/L (sensitivity 97%, specificity 97%);<br>120 minutes: > 29.3 nmol/L (sensitivity 100%, specificity 93.4%) |
| **Kim, 2020.**[23] | 120 suspected AI | • IV 250mcg AST<br>• Serum and salivary cortisol at 0, 30, 60 | Packard Cobra Gamma Counter analyzer RIA; | EIA (Salimetrics) | R=0.750, *P*<0.001 | Peak salivary cortisol >13.2 nmol/L (sensitivity 90.7%; specificity 94.1%). |

| | | | | | | |
|---|---|---|---|---|---|---|
| | | mins | serum cortisol >496.8 nmol/L | | | |
| **Kosák, 2014.**[27] | 84 suspected AI | • IV 250mcg AST<br>• Serum and salivary cortisol at 0, 30, 60 minutes | Radio-immunoassay; Peak cortisol > 500 nmol/L | Immunoassay | R=0.773, *P*<0.001 | Peak salivary cortisol >27.54 nmol/L (Sn/Sp not reported) |
| **Langelaan, 2018.**[19] | 129 suspected AI | • IV 250mcg AST<br>• Serum and salivary cortisol measured at 0, 30, 60 minutes | Roche immunoassay Cortisol 1: ≥ 550 nmol/L Cortisol 2: ≥ 420 nmol/L | LC-MS | NA | Peak salivary cortisol:<br>0 minutes: ≥5.9 nmol/L<br>30 minutes: ≥17.2 nmol/L<br>60 minutes: ≥15.6 nmol/L<br>(Sn/Sp not reported) |
| **Mak, 2017.**[20] | 56 healthy volunteers<br>171 suspected AI | • IV 1mcg AST<br>• Serum and salivary cortisol measured at 0, 30, 60 minutes | Abbott Architect immunoassay Peak serum cortisol ≥376 nmol/L | LC-MS | R=0.779 | Peak salivary cortisol ≥8.6 nmol/L (sensitivity 84.7%, specificity=94.6%) |
| **Marcus-Perlman, 2006.**[28] | 14 healthy volunteers, 14 women (pregnant or on OCP), 10 AI | • IV 1mcg AST<br>• Serum and salivary cortisol at 0, 30, 60 minutes | Roche immunoassay; peak cortisol > 496.8 nmol/L | Radio-immunoassay | R=0.74 | Serum cortisol at 30 minutes >24.28 nmol/L (Sn/Sp not reported) |
| **Nolan, 2017.**[21] | 24 healthy volunteers<br>10 healthy women on OCP<br>87 suspected AI | • IV 250mcg AST<br>• Serum and salivary cortisol measured at 0, 30, 60 minutes | Beckman DxI800 immunoassay; peak serum cortisol ≥500 nmol/L | LC-MS | NA | Peak salivary cortisol ≥26 nmol/L<br>(Sn/Sp not reported) |
| **Perogamvros, 2010.**[22] | 78 suspected AI | • IM 250mcg AST (n=68)<br>• ITT (n=10) | Siemens Centaur immunoassay; Peak serum cortisol >500 nmol/L | LC-MS | $R^2$=0.83, *P*<0.001 | Peak salivary cortisol >8.3 nmol/L (sensitivity 100%, specificity 100%) |

| | | | | | | |
|---|---|---|---|---|---|---|
| **Raff, 2008.**[33] | 28 hospitalised patients with low serum albumin, suspected AI | • IV 5mcg or 250mcg AST<br>• Serum and salivary cortisol at 30 minutes | Centaur immunoassay; serum cortisol > 497 nmol/L 30 minutes after AST | Enzyme-linked immunoassay | NA | NA |
| AI: adrenal insufficiency<br>OCP: oral contraceptive pills<br>EIA: enzyme immunoassay<br>LC-MS: liquid chromatography mass spectrometry | | | | | | |
